# Estimate of the hydraulic force in the aging heart: a cardiovascular magnetic resonance imaging study

**DOI:** 10.1186/s12880-024-01303-7

**Published:** 2024-07-08

**Authors:** Moussa Gueda Moussa, Jérôme Lamy, Vincent Nguyen, Perrine Marsac, Umit Gencer, Elie Mousseaux, Emilie Bollache, Nadjia Kachenoura

**Affiliations:** 1 Laboratoire d’Imagerie Biomédicale (LIB), Sorbonne Université, CNRS, INSERM, 15 Rue de École de Médecine, Paris, 75006 France; 2 PARCC, Université Paris Cité, Inserm, Hôpital Européen Georges Pompidou, Assistance Publique-Hôpitaux de Paris, Paris, France

**Keywords:** MRI, Left ventricle, Left atrium, Coupling, Aging

## Abstract

**Background:**

Coupling between left ventricle (LV) and left atrium (LA) plays a central role in the process of cardiac remodeling during aging and development of cardiac disease. The hydraulic force (HyF) is related to variation in size between LV and LA. The objectives of this study were to: (1) derive an estimate of left atrioventricular HyF using cine- Magnetic Resonance Imaging (MRI) in healthy subjects with a wide age range, and (2) study its relationship with age and conventional diastolic function parameters, as estimated by reference echocardiography.

**Methods:**

We studied 119 healthy volunteers (mean age 44 ± 17 years, 58 women) who underwent Doppler echocardiography and MRI on the same day. Conventional transmitral flow early (E) and late (A) LV filling peak velocities as well as mitral annulus diastolic longitudinal peak velocity (E’) were derived from echocardiography. MRI cine SSFP images in longitudinal two and four chamber views were acquired, and analyzed using feature tracking (FT) software. In addition to conventional LV and LA strain measurements, FT-derived LV and LA contours were further used to calculate chamber cross-sectional areas. HyF was approximated as the difference between the LV and LA maximal cross-sectional areas in the diastasis phase corresponding to the lowest LV-LA pressure gradient. Univariate and multivariate analyses while adjusting for appropriate variables were used to study the associations between HyF and age as well as diastolic function and strain indices.

**Results:**

HyF decreased significantly with age (R²=0.34, *p* < 0.0001). In addition, HyF was significantly associated with conventional indices of diastolic function and LA strain: E/A: R²=0.24, *p* < 0.0001; E’: R²=0.24, *p* < 0.0001; E/E’: R²=0.12, *p* = 0.0004; LA conduit longitudinal strain: R²=0.27, *p* < 0.0001. In multivariate analysis, associations with E/A (R^2^ = 0.39, *p* = 0.03) and LA conduit strain (R^2^ = 0.37, *p* = 0.02) remained significant after adjustment for age, sex, and body mass index.

**Conclusions:**

HyF, estimated using FT contours, which are primarily used to quantify LV/LA strain on standard cardiac cine MRI, varied significantly with age in association with subclinical changes in ventricular filling. Its usefulness in cohorts of patients with left heart disease to detect LV-LA uncoupling remains to be evaluated.

## Introduction

Aging is associated with left ventricular (LV) diastolic function impairment [1], an increase in myocardial fibrosis and a higher risk of atrial fibrillation [2, 3]. The LV and the left atrium (LA) are privileged targets of cardiovascular aging vicious circle. Among subclinical changes in LV and LA described in the setting of aging, we can identify volumetric modifications through LV shrinkage and LA enlargement [4, 5]. Such volumetric alterations may underlie changes in inner pressure gradients and hemodynamics such as changes in LV early and late filling velocities. Indeed, in the healthy state, LA and LV are working closely together in a harmonious anatomical and hemodynamic coupling regulating their filling and emptying phases throughout the cardiac cycle. In the setting of aging or disease, unbalanced changes in the LV or the LA may hinder such balanced coupling and ultimately induce further impairment of left heart chambers and myocardium. Cardiac magnetic resonance imaging (MRI) has emerged as a powerful tool for the assessment of cardiac chamber volumes [6], myocardial fibrosis, heart valve disease [7] and heart chamber strain, while using tagging images [8] or, more recently, cine MRI images along with feature tracking [9–13]. Most MRI feature tracking studies have focused on either LV systolic and diastolic function [14], or LA volumetric changes or tri-phasic function [15–18]. Only few studies investigated both the LA and LV, while reporting indices to assess their coupling, or uncoupling [19, 20]. Among these, geometric LA/LV volume ratio estimated from MRI and echocardiography has been proposed as an atrioventricular coupling index in elderly healthy subjects [21, 22] and in patients with heart failure [23]. In addition, the hemodynamic component of such coupling was described through the hydraulic force (HyF), which has been defined as a macroscopic contributor to the apico-basal longitudinal motion of the atrioventricular plane and a parallel mechanism to the molecular restoring forces generated by LV contraction, both favoring LV filling. HyF was first demonstrated to be the sole contributor to diastolic filling, using a mathematical rigid heart model, where the atrioventricular plane was treated as a piston with two adjacent compartments [24]. Despite this simplification, simulations replicated changes in pressure and flow over the cardiac cycle within physiological ranges, and suggested that this force would have a magnitude similar to other forces involved in ventricular filling. Inspired by this piston model, it was further shown that LV apex-to-base HyF could be interpreted as a consequence of left heart anatomy. Indeed, a first imaging study [24] hypothesized that the heart geometry favored the apico-basal motion of the atrioventricular annulus when LA cross-section is smaller than LV cross-section during diastolic filling, such as in a physiologically normal heart, and that such difference would drive the HyF magnitude. Literature studies illustrated the effects of HyF in an LA and LV analogous physical model, and then estimated HyF in vivo from atrial and ventricular MRI short-axis slices in a small population of young healthy volunteers [24] and then in patients with heart failure [25]. In the present study, we aimed to build on such literature and: (1) to take advantage of LV and LA contours defined by conventional feature tracking for strain assessment from cine MRI data to measure HyF between the LA and LV, (2) to evaluate HyF changes in healthy participants aged from 20 to 81 years, and (3) to evaluate associations between HyF and LV filling function, as assessed by reference echocardiographic measurements, performed on the same day as MRI.

## Materials and methods

### Study population

We retrospectively studied 121 healthy volunteers with age ranging from 20 to 81 years with equivalent distribution between men and women (mean age: 44.0 ± 16.5 years, 60 women). All subjects were asymptomatic and free of overt cardiovascular disease. Each volunteer underwent MRI and Doppler echocardiography on the same day. The study protocol was approved by the institutional review board and informed consent was obtained from all participants.

### Echocardiographic LV diastolic function parameters

Conventional LV diastolic function parameters were measured by a 15-year experienced echocardiographer using routine pulsed Doppler echocardiography on a GEMS Vivid 7 device (GE HealthCare, Chicago, IL, USA). These acquisitions provided transmitral flow peak velocities (E wave, cm/s: early LV filling peak velocity and A wave, cm/s: late LV filling peak velocity) as well as mitral annulus diastolic longitudinal peak velocity (E’, cm/s). E/A and E/E’ ratios were further calculated.

### MRI acquisitions and volumetric measurements

MRI exams were performed on a 1.5T magnet (GE HealthCare, Chicago, IL, USA). All participants underwent a full cardiac MRI exam, including long axis (two- and four-chamber views) and short axis steady-state free precession (SSFP) cine images, acquired with retrospective ECG gating during consecutive breath-holds. The short-axis acquisitions consisted of a stack of 10 to 14 slices covering the left ventricle from its base to apex. Average acquisition parameters were: acquired spatial resolution 0.7 × 0.7 mm², slice thickness 7 to 8 mm, acquisition matrix 224 × 192, repetition time 3.5 ms, echo time 1.5 ms, and flip angle 50°. Cine data were reconstructed into 30 to 60 frames per cardiac cycle. All cine images were viewed and rated in terms of image quality and presence of artifacts. Blood pressures were measured for all participants immediately after the MRI exam. Medis Suite MR Software (version 6, Medis Medical Imaging, Leiden, the Netherlands) was used to estimate LV and LA volumes. LV volumes were calculated from the stack of short axis images and LA volumes were estimated using the biplane area-length method [17] from 2- and 4-chamber views. LV end-systolic (LV ESV), LV end-diastolic (LV EDV) volumes, and LV mass, as well as LA end-systolic (LA ESV) and LA end-diastolic (LA EDV) volumes were reported. LV and LA ejection fractions (LVEF and LAEF, respectively) were further calculated. Volumetric parameters were indexed to body surface area (BSA).

### MRI strain analysis

LV and LA strain analyses were performed using CardioTrack software (LIB, Sorbonne University) [5, 26–28] by an operator blinded to the abovementioned volumetric measurements. This software was previously validated against myocardial histological findings in patients undergoing mitral valve surgery [29] and was shown to be reproducible for all heart chambers in both inter-operator and scan–rescan evaluations [13]. First, LV epicardial and endocardial contours were manually traced on both long axis and short axis images, on a single phase corresponding to maximal LV dilation to initialize tracking throughout the cardiac cycle. LA endocardial contour was traced on a single temporal phase corresponding to maximum LA dilation on two- and four-chamber views while excluding pulmonary veins from LA cavity [5, 26]. After automated tracking throughout all phases of the cardiac cycle, LV global longitudinal strain (GLS) as well as global circumferential (GCS) and radial (GRS) strain were estimated on the long axis and short axis views, respectively. LA longitudinal strain (SL) was estimated while averaging values obtained on 2- and 4-chamber views, and LA triphasic function was quantified through reservoir (SL_R_), conduit (SL_C_), and booster (SL_B_) strain values.

### Estimation of hydraulic force from MRI feature tracking contours

**Fig. 1:**
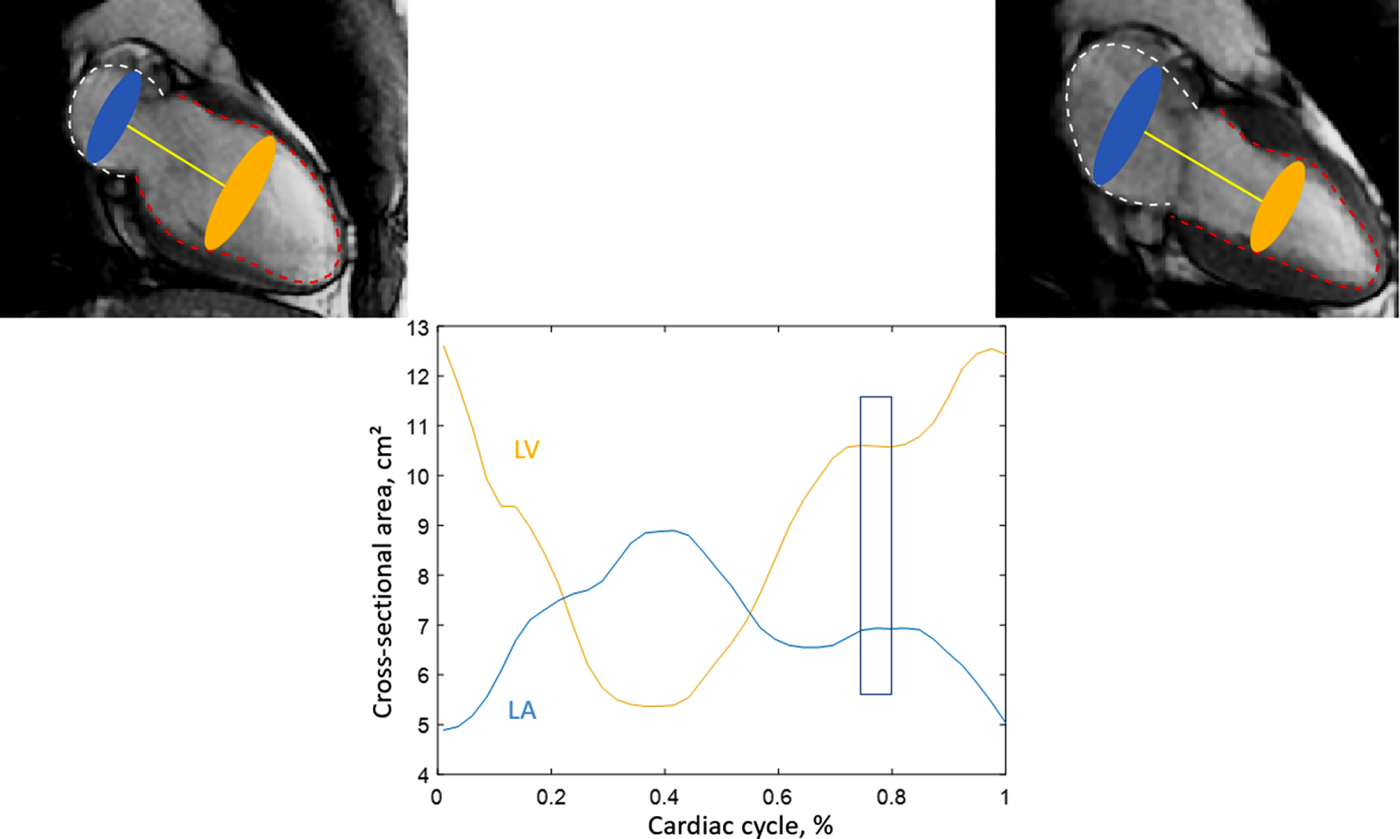
Estimation of time-resolved left atrial and left ventricular maximal transverse cross-sectional areas. Upper row: transverse areas are illustrated on the end-diastolic (left) and end-systolic (right) phases for left atrium (blue) and left ventricule (orange). Bottom row: left atrium (blue) and left ventricle (orange) cross-sectional area curves during the cardiac cycle, where diastasis phase is highlighted by the blue box

LV and LA cross-sectional areas were estimated from feature tracking-derived endocardial contours at LV and LA maximal transverse dimension, respectively, perpendicular to the long axis connecting their centers of mass (Fig. 1), for all phases of the cardiac cycle. Then, the HyF was estimated as the difference in left atrioventricular cross-sectional areas, as proposed by Maksuti et al. [24]: HyF (cm²) = LV cross-sectional area - LA cross-sectional area. HyF was quantified in the diastasis phase to minimize the effect of pressure differences between the LA and the LV and thus neglect the pressure component.

### Statistical analysis

Volunteers were subdivided into two age groups: G1 < 50 and G2 ≥ 50 years. Continuous variables are presented as means ± standard deviations (SD). Differences between G1 and G2 age groups were tested with a non-parametric Wilcoxon test. Boxplot graphs were also used to illustrate differences between groups. Associations with age were studied using linear regressions in univariate analysis. Moreover, univariate associations of HyF with conventional echocardiographic parameters of diastolic function and strain assessed by MRI were studied using linear regressions, and adjustment for age, body mass index (BMI) and sex was performed in multivariate models. R² Pearson correlation coefficients and linear regression equation were provided. HyF intra- and inter-observer reproducibility was evaluated in 20 randomly selected subjects and intraclass correlation coefficient (ICC) was provided. For such analysis, measurements were performed by two independent operators (MGM, PM), and operator 1 repeated analysis one month after the first evaluation. ICC was considered as excellent (> 0.74), good (0.60–0.74), moderate (0.40–0.59), or poor (< 0.40) [30]. All tests with a P-value < 0.05 were considered statistically significant. Statistical analyses were performed using JMP software (version 15, SAS Institute, Cary, NC, USA).

## Results

Two datasets were excluded due to poor image quality or presence of artefacts (n = 2 women). Baseline characteristics, central pressures, as well as MRI LV and LA volumetric measurements of the remaining 119 healthy volunteers are summarized in Table 1. There was a significant increase in systolic and diastolic blood pressures as well as BMI in the elderly as compared to the younger group (p ≤ 0.01). LV echocardiographic diastolic function indices were significantly different between the two age groups, as revealed by a decrease in E, E/A, E’ as well as an increase in A and E/E’ with age (*p* < 0.0001). Moreover, we found a significant decrease in indexed LV volumes and increase in indexed LA volumes with age (*p* ≤ 0.02). Finally, there was no significant difference in LA, LV ejection fraction and indexed LV mass between the 2 age groups.

**Table 1:** Study population basic characteristics along with echocardiographic diastolic function and MRI volumetric measurements

	G1 (<50 years)	G2 (≥50 years)	*P* value
*N*	72	47	
Age (years)	32.5 ± 8.4	61.3 ± 8.6	<0.0001
Men/women (n)	35/37	26/21	ns
BMI (kg/m^2^)	22.9 ± 3.0	24.5 ± 3.4	0.01
BSA (m²)	1.76 ± 0.2	1.82 ± 0.2	ns
HR (bpm)	69.7 ± 10.3	67.1 ± 8.7	ns
Brachial SBP (mmHg)	107.9 ± 8.1	117.9 ± 12.1	<0.0001
Brachial DBP (mmHg)	66.3 ± 8.3	72.7 ± 7.9	<0.0001
**Echocardiographic measurements**			
E (cm/s)	80.2 ± 16.8	66.6 ± 11.6	<0.0001
A (cm/s)	55.5 ± 11.8	71.1 ± 15.1	<0.0001
E/A	1.5 ± 0.4	0.98 ± 0.3	<0.0001
E’ (cm/s)	16.9 ± 9.3	10.1 ± 2.5	<0.0001
E/E’	4.9 ± 1.2	7.0 ± 1.7	<0.0001
**MRI volumetric measurements**			
LV mass_i_ (g/m^2^)	59.5 ± 10.5	59.2 ± 12.2	ns
LV EDVi (ml/m^2^)	74.7 ± 14.8	66.9 ± 16.9	0.0006
LV ESVi (ml/m^2^)	27.7 ± 7.8	24.3 ± 6.8	0.003
LV EF (%)	63.3 ± 5.5	63.6 ± 5.1	ns
LA EDVi (ml/m^2^)	24.7 ± 6.8	28.9 ± 10.0	0.02
LA ESVi (ml/m^2^)	11.0 ± 3.3	13.9 ± 4.7	0.001
LA EF (%)	55.8 ± 7.4	55.4 ± 6.3	ns

BMI: body mass index, BSA: body surface area, HR: heart rate measured during MRI, SBP/DBP: systolic/diastolic blood pressure, E: transmitral early maximal velocity, A: transmitral late maximal velocity, E’: mitral annulus maximal longitudinal velocity, LV: left ventricle, LA: left atrium, Mass_i_: indexed mass, EDVi: end-diastolic volume index, ESVi: end-systolic volume index, EF: ejection fraction, ns: non-significant

### Conventional LV and LA strain indices

MRI feature tracking-derived LV and LA strain indices are summarized in Table 2. LV global longitudinal (GLS) and circumferential (GCS) strain remained unchanged between the two age groups, while there was a slight decrease in LV radial strain (GRS) with age. LA reservoir and conduit longitudinal strain were significantly lower in the elderly subjects as compared to younger subjects (*p* < 0.0001). The LA booster longitudinal strain slightly increased in the elderly but such elevation did not reach statistical significance.

**Table 2:** Quantitative measurements estimated using feature tracking from MRI cine data

	G1(<50 years)	G2 (≥50 years)	*P* value	Correlation coefficient with age (*R*²)
**Left ventricle (LV)**				
LV GLS (%)	−16.9 ± 2.8	−16.0 ± 3.4	ns	–
LV GCS (%)	−18.3 ± 2.7	−17.6 ± 2.7	ns	–
LV GRS (%)	57.2 ± 12.5	52.1 ± 13.7	0.02	0.04
**Left atrium (LA)**				
LA SL_R_ (%)	30.2 ± 5.9	24.1 ± 4.9	<0.0001	0.27
LA SL_C_ (%)	18.5 ± 3.8	11.8 ± 3.9	<0.0001	0.54
LA SL_B_ (%)	11.8 ± 5.0	12.2 ± 2.9	ns	–
**Diastasis areas and HyF**				
LV cross-sectional area (cm²)	12.9 ± 4.0	10.9 ± 4.9	0.01	0.08
LA cross-sectional area (cm²)	7.9 ± 2.7	9.7 ± 2.9	0.0005	0.08
HyF (cm²)	4.9 ± 2.9	1.2 ± 3.4	<0.0001	0.34

LV: left ventricle, LA: left atrium, GLS: global longitudinal strain, GCS: global circumferential strain, GRS: global radial strain, SL_R_: reservoir longitudinal strain, SL_C_: conduit longitudinal strain, SL_B_: booster longitudinal strain, HyF: hydraulic force, ns: non-significant

### Left atrioventricular coupling: hydraulic force

**Fig. 2:**
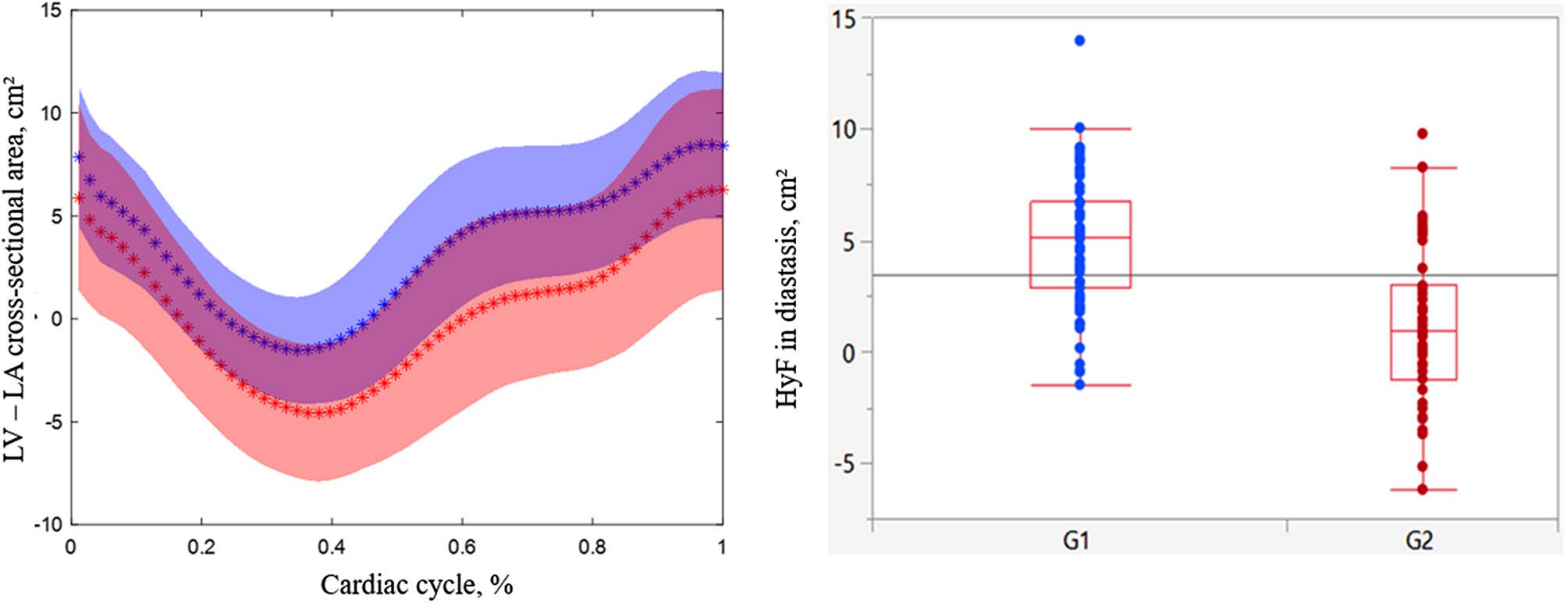
Left ventricular – left atrial (LV-LA) cross-sectional area difference in younger and elderly healthy volunteers. LV-LA cross-sectional area difference averaged over younger (blue) and elderly (red) healthy volunteers during the cardiac cycle (left) and values according to age group during diastasis which estimate hydraulic force (right). Lines indicated by ‘*’ symbols correspond to the mean and shaded zones illustrate standard deviation

**Fig. 3:**
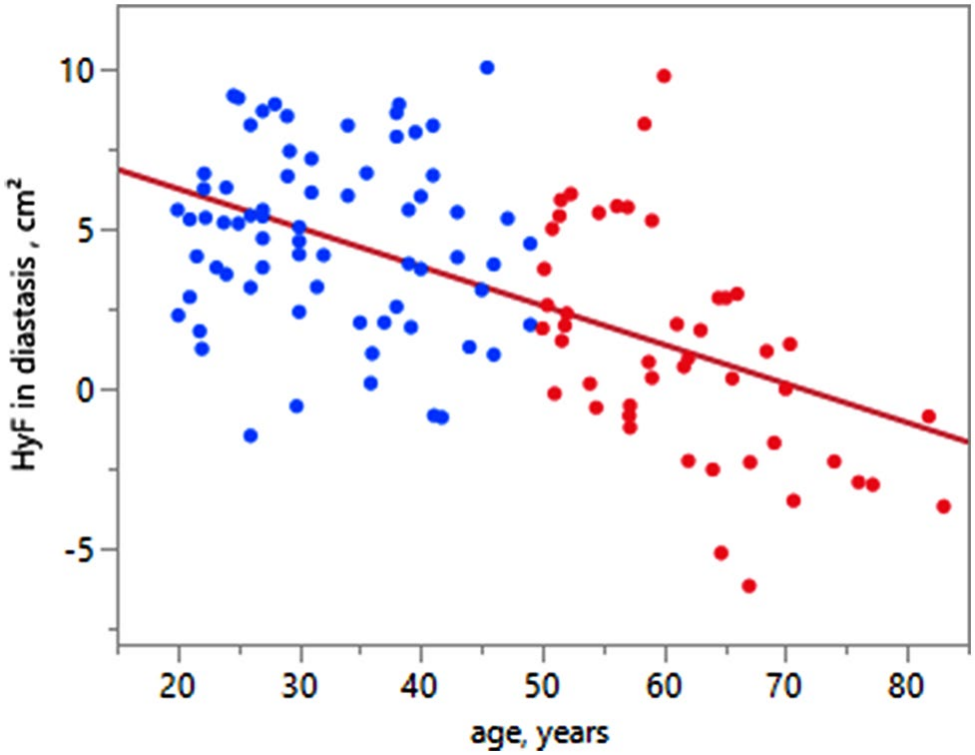
Linear regression for the association of hydraulic force measured from MRI with age. Younger and elderly healthy volunteers are depicted in blue and red, respectively

**Fig. 4:**
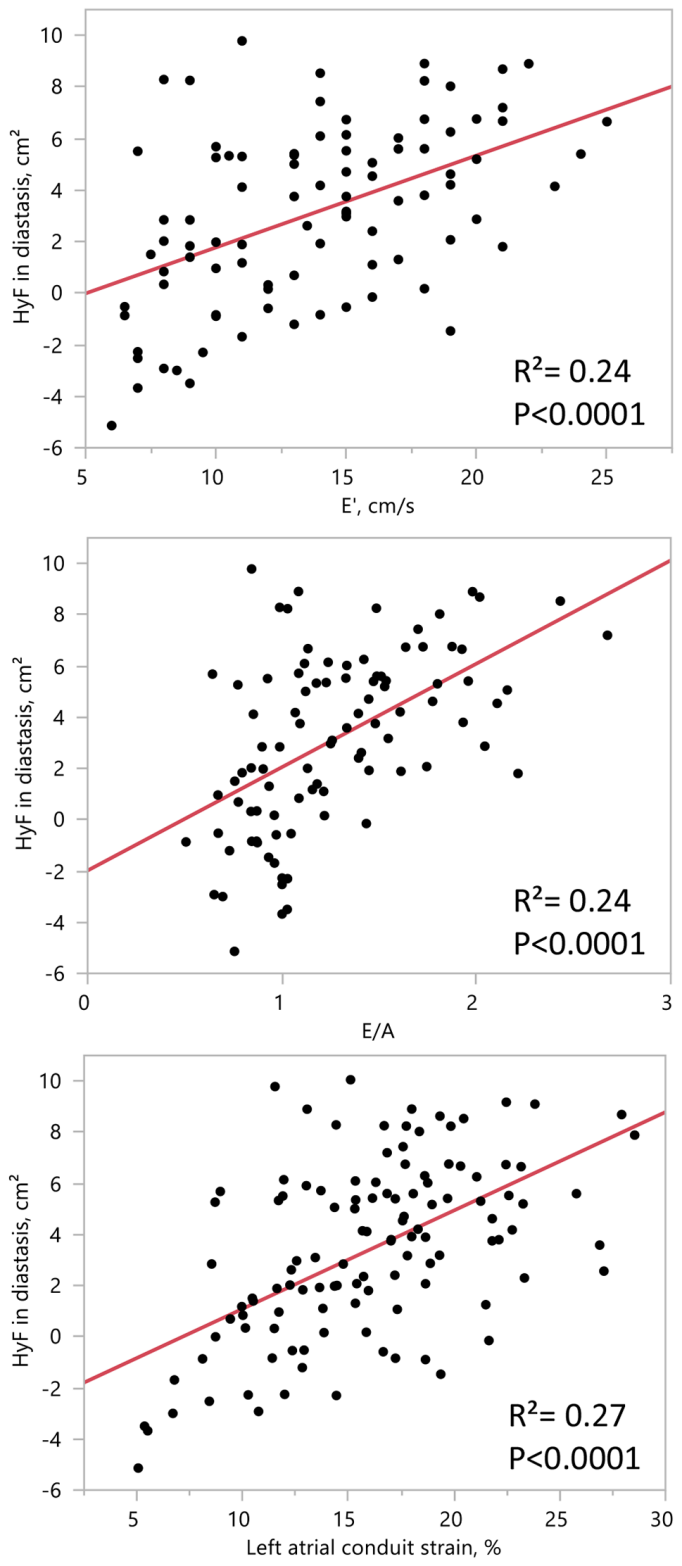
Linear regressions of hydraulic force measured from MRI with echocardiographic diastolic function parameters and left atrial conduit strain. Association with E’ (upper row), E/A (middle row) and left atrial conduit strain (bottom row)

Time-resolved HyF averaged over the two age groups is illustrated in Fig. 2, highlighting a more pronounced difference between age groups during the diastasis phase. Similar to volumetric changes with age, LV transverse cross-sectional area decreased significantly while LA transverse cross-sectional area increased significantly with age, resulting in a significant age-related decrease in HyF (Tables 2 and Fig. 3). Furthermore, significant associations were found between HyF and echocardiographic diastolic function parameters (E/A, E’ and E/E’) as well as LA conduit function strain (Table 3). The strongest associations between HyF and echocardiographic diastolic function parameters are illustrated in Fig. 4. Associations with echocardiographic transmitral E/A (R^2^ = 0.39, *p* = 0.03) and atrial conduit strain (R^2^ = 0.37, *p* = 0.02) remained significant after adjustment for age, BMI and sex (Table 3).

**Table 3:** Associations of hydraulic force with conventional echocardiographic diastolic function parameters and LA conduit strain

Variables	Correlation coefficient (*R*²)	*P*-value
E’	0.24	<0.0001
E/A	0.24*	<0.0001
E/E’	0.12	0.0004
LA SL_C_	0.27*	<0.0001

LA: left atrial, SL_C_: conduit longitudinal strain, E: transmitral early maximal velocity, A: transmitral atrial late maximal velocity and E’: mitral annulus maximal longitudinal velocity

*: associations remained significant after adjustment to age, BMI and sex

### Intra- and inter-observer reproducibility

Intra- and inter-operator reproducibility of HyF measurement was excellent as revealed by intra-operator ICC of 0.82 and inter-operator ICC of 0.80, respectively.

## Discussion

In the present study, hydraulic force was estimated in addition to LV and LA strain indices with excellent intra- and inter-operator reproducibility, using feature tracking contours obtained on standard-of-care cardiac cine SSFP MRI images. Consistency of our feature tracking processing algorithm is highlighted by the associations between LA strains and age that are in line with previous literature [5, 31–34]. As a contributor to LV filling, the estimated HyF was able to detect age-related subclinical alteration in LV function, and was associated with MRI-independent and strain-derived indices of LV filling, namely echocardiographic E/A ratio and MRI LA conduit strain, respectively.

LV and LA are intimately coupled during the diastolic phase through pressure gradients and blood flowing through the opened transmitral valve. Age, along with underlying geometrical remodeling unbalance the harmonious and pressure-regulated interaction between the two left heart chambers. Indeed, several studies have highlighted subclinical variations in LA and LV volumes with age [35–38] along with alterations in LV filling pattern [39–41], as assessed by both echocardiography and MRI, although ejection fraction of both chambers remains frequently preserved. An unbalanced LV and LA coupling, as quantified by the LA to LV volumes ratio, has been shown to be predictive of adverse cardiovascular events [19, 21, 23]. Accordingly, distinguishing physiological from pathological variations of the LV and LA interactions requires a priori knowledge of expected physiological age-related subclinical changes in asymptomatic individuals.

Analysis of cardiovascular dynamic images allows, through readily available segmentation tools, to quantify heart chamber remodeling, their myocardial strain, as well as LV diastolic function through specific velocity-based acquisitions, focusing on transmitral flow. Accordingly, a recent MRI review summarized conventional measurement normative values and expected age-related variations [42] in both adult and pediatric populations. Beyond volumetric measurements and strain, heart time-resolved segmentation provided by feature tracking can be used to further investigate the coupling between the LV and the LA throughout the cardiac cycle, without extra acquisition or processing efforts. Among indices of left heart coupling relying on chambers segmentation, investigators previously proposed LA to LV volume ratio from both echocardiography [22] and MRI [21] images. Another parameter, which has been described as a contributor to diastolic filling is the hydraulic force, which was previously reported in an experimental model and a small group of healthy volunteers (*n* = 10) [24]. The present study builds on these reported results of the hydraulic force and expands to a large group of asymptomatic individuals with a wide age range and MRI-independent measurement of diastolic function.

Myocardial recoil during early LV filling induces a drop in intraventricular pressure and a subsequent blood flow from the LA towards the LV. During such LV filling, the cross-sectional area of the LV is increased and the hydraulic force will further promote filling by pushing the mitral annulus towards the atrium. In elderly subjects, such recoil tends to diminish, while myocardium stiffens concomitantly to changes in LV and LA volumes, as LA dilates [37, 43] and LV shrinks [35, 44] with age, affecting the direction and magnitude of the hydraulic force. After 50 years, LA cross-sectional area tends to equalize or even exceed LV cross-sectional area, reducing hydraulic force magnitude, and thus its contribution to LV filling. One might note the larger HyF standard deviation around mean value in elderly compared to younger individuals, highlighting heterogeneous age-related remodeling of left heart chambers in individuals above 50 years. The drop in HyF with age is in line with previous findings in heart failure with preserved ejection fraction [25]. The independent associations found in our study between HyF and E/A ratio index as well as LA conduit strain further confirm the contributing role of HyF to LV filling.

One limitation of the present study is the absence of patients with cardiomyopathies to fully demonstrate the clinical usefulness of HyF. However, our primary goal was to evaluate the contributing role of HyF to LV filling in the pathway of normal aging. Our study highlighted that HyF can be easily derived from feature tracking contours with high reproducibility to warrant upcoming studies in patients, as feature tracking is applied in various disease conditions nowadays. Another limitation is that HyF was estimated in our study without accounting for intra-LA and LV pressures. However, one might highlight that HyF was quantified during diastasis when LA-LV pressures nearly equalize. Obtaining such measurements would require invasive procedure, which is not conceivable in healthy cohorts.

## Conclusion

The intricated relationship between LA and LV geometry through age can be easily depicted through quantitative indices such as hydraulic forces that can be estimated from feature tracking contours, conventionally used in standard cardiac MRI for strain estimation, without extra scan and analysis time. Such index was able to depict age-related subclinical alterations in LV filling. Its usefulness in cohorts of patients with left heart disease to reveal LV-LA deleterious coupling remains to be evaluated.

## Data Availability

Data that support the findings of this study are available on request from the corresponding author.

## Abbreviations

LA: Left atrium
LV: Left ventricle
HyF: Hydraulic force
MRI: Magnetic Resonance Imaging
FT: Feature tracking
SSFP: steady-state free precession
ESV: End-systolic volume
EDV: End-diastolic volume
EF: Ejection fraction
ICC: Intraclass correlation coefficient
